# Supplementary material for: The diagnostic performance of CA125 for the detection of ovarian and non-ovarian cancer in primary care: A population-based cohort study
Source: PLoS Med. 2020 Oct 28;17(10):e1003295. doi: 10.1371/journal.pmed.1003295 (PMC7592785; doi:10.1371/journal.pmed.1003295)
Supplement: S1 Text — ISAC, Independent Scientific Advisory Committee. (PDF) [file pmed.1003295.s001.pdf]

ISAC APPLICATION FORM

PROTOCOLS FOR RESEARCH USING THE CLINICAL PRACTICE RESEARCH DATALINK (CPRD)

For ISAC use only		
Protocol No.<br><br>Submission date<br>(DD/MM/YYYY)	...........................<br><br>...........................	***IMPORTANT***<br>*Please refer to the* ***guidance*** *for '****Completing the ISAC application form'*** *found on the CPRD website (www.cprd.com/isac). If you have any queries, please contact the ISAC Secretariat at isac@cprd.com.*

SECTION A: GENERAL INFORMATION ABOUT THE PROPOSED RESEARCH STUDY

1. Study Title§ (*Please state the study title below)*

The role of CA125 in the detection of ovarian cancer in symptomatic primary care patients

§Please note: This information will be published on the CPRD's website as part of its transparency policy.

2. Has any part of this research proposal or a related proposal been previously submitted to ISAC?

Yes* ☐ No ☒

**If yes, please provide the previous protocol number/s below. Please also state in your current submission how this/these are related or relevant to this study.*

3. Has this protocol been peer reviewed by another Committee? (e.g. grant award or ethics committee)

Yes* ☐ No ☒

**If Yes, please state the name of the reviewing Committee(s) below and provide an outline of the review process and outcome as an Appendix to this protocol :*

4. Type of Study (please tick all the relevant boxes which apply)

Adverse Drug Reaction/Drug Safety	☐	Drug Effectiveness	☐
Drug Utilisation	☐	Pharmacoeconomics	☐
Disease Epidemiology	☒	Post-authorisation Safety	☐
Health care resource utilisation	☒	Methodological Research	☐
Health/Public Health Services Research	☒	Other*	☒

**If Other, please specify the type of study here and in the lay summary below:*
Test diagnostic accuracy study

5. Health Outcomes to be Measured§

§Please note: This information will be published on CPRD's website as part of its transparency policy.

Please summarise below the primary/secondary health outcomes to be measured in this research protocol:

Primary outcome:
- Ovarian cancer diagnosis

Secondary outcomes:
- Diagnosis of a cancer other than ovarian
- Histological type and morphology, stage, size, grade of ovarian cancer at diagnosis
- Death from ovarian cancer or death from another cancer

6. Publication: This study is intended for (please tick all the relevant boxes which apply)**:**

Publication in peer-reviewed journals ☒ Presentation at scientific conference ☒
Presentation at company/institutional meetings ☒ Regulatory purposes ☐
Other* ☐

**If Other, please provide further information:*

SECTION B: INFORMATION ON INVESTIGATORS AND COLLABORATORS

7. Chief Investigator§
Please state the full name, job title, organisation name & e-mail address for correspondence - see guidance notes for eligibility. Please note that there can only be one Chief Investigator per protocol.

Dr Fiona Walter, Principal Researcher in Primary Care Cancer Research, University of Cambridge, fmw22@medschl.cam.ac.uk

§Please note: The name and organisation of the Chief Investigator and will be published on CPRD's website as part of its transparency policy

CV has been previously submitted to ISAC ☐ **CV number:**
A new CV is being submitted with this protocol ☐
An updated CV is being submitted with this protocol ☒

8. Affiliation of Chief Investigator (full address)
Primary Care Unit
Department of Public Health and Primary Care
Strangeways Research Laboratory
2 Worts' Causeway
Cambridge CB1 8RN

9. Corresponding Applicant§
Please state the full name, affiliation(s) and e-mail address below:
Dr Garth Funston, Department of Public Health and Primary Care, University of Cambridge, gf272@cam.ac.uk
§Please note: The name and organisation of the corresponding applicant and their organisation name will be published on CPRD's website as part of its transparency policy

Same as chief investigator ☐
CV has been previously submitted to ISAC ☒ **CV number:** 234_18
A new CV is being submitted with this protocol ☐
An updated CV is being submitted with this protocol ☐

10. List of all investigators/collaborators§
Please list the full name, affiliation(s) and e-mail address* of all collaborators, other than the Chief Investigator below:

§Please note: The name of all investigators and their organisations/institutions will be published on CPRD's website as part of its transparency policy

Other investigator: Professor Willie Hamilton,
Exeter Medical School, University of Exeter, w.hamilton@exter.ac.uk
CV has been previously submitted to ISAC ☒ **CV number:** 201_15CEPS
A new CV is being submitted with this protocol ☐
An updated CV is being submitted with this protocol ☐

Other investigator: Dr Emma Crosbie
Department of Gynaecological Oncology, University of Manchester, emma.crosbie@manchester.ac.uk
CV has been previously submitted to ISAC ☐ **CV number:**
A new CV is being submitted with this protocol ☒
An updated CV is being submitted with this protocol ☐

Other investigator: Dr Gary Abel
Exeter Medical School, University of Exeter, g.a.abel@exeter.ac.uk

CV has been previously submitted to ISAC	☒	**CV number:**	149_18
A new CV is being submitted with this protocol	☐		
An updated CV is being submitted with this protocol	☐		

Other investigator:

CV has been previously submitted to ISAC	☐	**CV number:**	
A new CV is being submitted with this protocol	☐		
An updated CV is being submitted with this protocol	☐		

[Please add more investigators as necessary]

Please note that your ISAC application form and protocol **must be copied to all e-mail addresses listed above at the time of submission of your application to the ISAC mailbox. Failure to do so will result in delays in the processing of your application.*

11. Conflict of interest statement*

Please provide a draft of the conflict (or competing) of interest (COI) statement that you intend to include in any publication which might result from this work

We have no conflicts of interest to declare.

**Please refer to the International Committee of Medical Journal Editors (ICMJE) for guidance on what constitutes a COI.*

12. Experience/expertise available

Please complete the following questions to indicate the experience/ expertise available within the team of investigators/collaborators actively involved in the proposed research, including the analysis of data and interpretation of results.

	**Previous GPRD/CPRD Studies**	**Publications using GPRD/CPRD data**
None	☐	☐
1-3	☐	☐
> 3	☒	☒

**Experience/Expertise available**	**Yes**	**No**
**Is statistical expertise available within the research team?**<br>*If yes, please indicate the name(s) of the relevant investigator(s)*<br>Dr Gary Abel	☒	☐
**Is experience of handling large data sets (>1 million records) available within the research team?**<br>*If yes, please indicate the name(s) of the relevant investigator(s)*<br>Professor Willie Hamilton, Dr Gary Abel	☒	☐
**Is experience of practising in UK primary care available to or within the research team?**<br>*If yes, please indicate the name(s) of the relevant investigator(s)*<br>Dr Garth Funston, Dr Fiona Walter, Professor Willie Hamilton	☒	☐

13. References relating to your study

Please list up to 3 references (most relevant) relating to your proposed study:

1) National Institute of Clinical Excellence. Suspected cancer: recognition and referral. www.nice.org.uk/guidance/ng12. Accessed 26 Jul 2017.
2) Hippisley-Cox J, Coupland C. Identifying women with suspected ovarian cancer in primary care: derivation and validation of an algorithm. BMJ. 2011;344:d8009–d8009.
3) Usher-Smith JA, Sharp SJ, Griffin SJ. The spectrum effect in tests for risk prediction, screening, and diagnosis. BMJ. doi: 10.1136/bmj.i3139

SECTION C: ACCESS TO THE DATA

14. Financial Sponsor of study§

§Please note: The name of the source of funding will be published on CPRD's website as part of its transparency policy

Pharmaceutical Industry	☐	*Please specify name and country:*
Academia	☐	*Please specify name and country:*
Government / NHS	☐	*Please specify name and country:*
Charity	☒	*Please specify name and country:* Cancer Research UK
Other	☐	*Please specify name and country:*
None	☐	

15. Type of Institution conducting the research

Pharmaceutical Industry	☐	*Please specify name and country:*
Academia	☒	*Please specify name and country:* University of Cambridge, UK
Government Department	☐	*Please specify name and country:*
Research Service Provider	☐	*Please specify name and country:*
NHS	☐	*Please specify name and country:*
Other	☐	*Please specify name and country:*

16. Data access arrangements

The financial sponsor/ collaborator* has a licence for CPRD GOLD and will extract the data	☐
The institution carrying out the analysis has a licence for CPRD GOLD and will extract the data**	☒
A data set will be provided by the CPRD¥€	☒
CPRD has been commissioned to extract the data and perform the analyses€	☐
Other:	☐

If Other, please specify:

**Collaborators supplying data for this study must be named on the protocol as co-applicants.*
***If data sources other than CPRD GOLD are required, these will be supplied by CPRD*
¥Please note that datasets provided by CPRD are limited in size; applicants should contact CPRD (enquiries@cprd.com) if a dataset of >300,000 patients is required.
€Investigators must discuss their request with a member of the CPRD Research team before submitting an ISAC application. Please contact the CPRD Research Team on +44 (20) 3080 6383 or email (enquiries@cprd.com) to discuss your requirements. Please also state the name of CPRD Research team with whom you have discussed this request (provide the date of discussion and any relevant reference information):

Name of CPRD Researcher Helen Booth Reference number (where available) Date of contact 1/3/18

17. Primary care data

Please specify which primary care data set(s) are required)

Vision only (Default for CPRD studies ☒ Both Vision and EMIS®* ☐
EMIS® only* ☐

Note: Vision and EMIS are different practice management systems. CPRD has traditionally collected data from Vision practice. Data collected from EMIS is currently under evaluation prior to wider release.
Investigators requiring the use of EMIS data **must discuss the study with a member of the CPRD Research team before submitting an ISAC application*

Please state the name of the CPRD Researcher with whom you have discussed your request for EMIS data:
Name of CPRD Researcher Reference number (where available) Date of contact

18. Site Location of Data

a) Processing location(s):

Location area - UK / EEA / Worldwide:

UK

Organisation address:

Primary Care Unit
Department of Public Health and Primary Care
Strangeways Research Laboratory
2 Worts' Causeway
Cambridge CB1 8RN
Note: Please enter the location details of where the data for this study will be used (processed).

b) Storage Location(s)

Location area - UK / EEA / Worldwide:

UK

Organisation address:
Primary Care Unit
Department of Public Health and Primary Care
Strangeways Research Laboratory
2 Worts' Causeway
Cambridge CB1 8RN
Note: Please enter the location details of where the data for this study will be stored.

c) Territory of analysis - UK / EEA / Worldwide:
UK

Primary Care Unit
Department of Public Health and Primary Care
Strangeways Research Laboratory
2 Worts' Causeway
Cambridge CB1 8RN

Note: Please enter the details of where the data for this study will be analysed.

SECTION D: INFORMATION ON DATA LINKAGES

19. Does this protocol seek access to linked data

Yes* ☒ No☐ If No, please move to section E.

Research groups which have not previously accessed CPRD linked data resources **must discuss access to these resources with a member of the CPRD Research team, before submitting an ISAC application. Investigators requiring access to HES Accident and Emergency data, HES Diagnostic Imaging Dataset, PROMS data, the Pregnancy Register, Cancer Registration, SACT and CPES data and the Mental Health Services Data Set **must** also discuss this with a member of the CPRD Research team before submitting an ISAC application. Please contact the CPRD Research Team on +44 (20) 3080 6383 or email enquiries@cprd.comto discuss your requirements **before** submitting your application.*

Please state the name of the CPRD Researcher with whom you have discussed your linkage request.

Name of CPRD Researcher Helen Booth Reference number (where available) Date of contact 1/3/18

Please note that as part of the ISAC review of linkages, your protocol may be shared - in confidence - with a representative of the requested linked data set(s) and summary details may be shared - in confidence - with the Confidentiality Advisory Group of the Health Research Authority.

20. Please select the source(s) of linked data being requested§
§Please note: This information will be published on the CPRD's website as part of its transparency policy.

☒ ONS Death Registration Data
☒ HES Admitted Patient Care
☐ HES Outpatient
☐ HES Accident and Emergency
☒NCRAS (National Cancer Registration and Analysis Service) Cancer Registration Data *
☐ NCRAS Cancer Patient Experience Survey (CPES) data*
☐ NCRAS Systemic Anti-Cancer Treatment (SACT) data*

☐ HES Diagnostic Imaging Dataset
☐ HES PROMS (Patient Reported Outcomes Measure)**
☐ CPRD Mother Baby Link
☐ Pregnancy Register

☐ Mental Health Services Data Set (MHDS)

☐ Practice Level Index of Multiple Deprivation (Standard)
☐ Practice Level Index of Multiple Deprivation (Bespoke)
☐ Patient Level Index of Multiple Deprivation***
☒ Patient Level Townsend Score ***

Applicants seeking access to NCRAS data must complete a Cancer Dataset Agreement form (available from CPRD). This should be submitted to the ISAC as an appendix to your protocol. Please also note that applicants seeking access to cancer registry data must provide consent for publication of their study title and study institution on the UK Cancer Registry website.
***Assessment of the quality of care delivered to NHS patients in England undergoing four procedures: hip replacement, knee replacement, groin hernia and varicose veins. Please note that patient level PROMS data are only available for non-commercial purposes, such as academic research, or in connection with delivering services to the NHS.*
**** 'Patient level IMD and Townsend scores will not be supplied for the same study*
*****If "Other" is specified, please provide the name of the individual in the CPRD Research team with whom this linkage has been discussed.*

Name of CPRD Researcher   Reference number (where available)   Date of contact

21. Total number of linked datasets requested <u>including</u> CPRD GOLD

Number of linked datasets requested (*practice/ 'patient' level Index of Multiple Deprivation, Townsend Score, the CPRD Mother Baby Link and the Pregnancy Register should **not** be included in this count)* 4

Please note: Where ≥5 linked datasets are requested, approval may be required from the Confidentiality Advisory Group (CAG) to access these data

22. Is linkage to a <u>local[¥]</u> dataset with <1 million patients being requested?

Yes* ☐   No ☒

**If yes, please provide further details:*
[¥] *Data from defined geographical areas i.e. non-national datasets.*

23. If you have requested one or more linked data sets, please indicate whether the Chief Investigator or any of the collaborators listed in question 5 above, have access to these data in a patient identifiable form (e.g. full date of birth, NHS number, patient post code), or associated with an identifiable patient index.

Yes* ☐   No ☒

** If yes, please provide further details:*

24. Does this study involve linking to patient *identifiable* data (e.g. hold date of birth, NHS number, patient post code) from other sources?

Yes ☐   No ☒

SECTION E: VALIDATION/VERIFICATION

25. Does this protocol describe a purely observational study using CPRD data?

Yes* ☒   No** ☐

** Yes: If you will be using data obtained from the CPRD Group, this study does not require separate ethics approval from an NHS Research Ethics Committee.*
*** No: You may need to seek separate ethics approval from an NHS Research Ethics Committee for this study. The ISAC will provide advice on whether this may be needed.*

26. Does this protocol involve requesting any additional information from GPs?

Yes* ☐ No ☒

** If yes, please indicate what will be required:*

Completion of questionnaires by the GP[Ψ]	Yes ☐	No ☐
Is the questionnaire a validated instrument?	Yes ☐	No ☐
If yes, has permission been obtained to use the instrument?	Yes ☐	No ☐
Please provide further information:		

Other (please describe)

[Ψ] Any questionnaire for completion by GPs or other health care professional must be approved by ISAC before circulation for completion.

27. Does this study require contact with patients in order for them to complete a questionnaire?

Yes* ☐ No ☒

**Please note that any questionnaire for completion by patients must be approved by ISAC before circulation for completion.*

28. Does this study require contact with patients in order to collect a sample?

Yes* ☐ No ☒

** Please state what will be collected:*

SECTION F: DECLARATION

29. Signature from the Chief Investigator

- I have read the guidance on '***Completion of the ISAC application form***' and '***Contents of CPRD ISAC Research Protocols***' and have understood these;
- I have read the submitted version of this research protocol, including all supporting documents, and confirm that these are accurate.
- I am suitably qualified and experienced to perform and/or supervise the research study proposed.
- I agree to conduct or supervise the study described in accordance with the relevant, current protocol
- I agree to abide by all ethical, legal and scientific guidelines that relate to access and use of CPRD data for research
- I understand that the details provided in sections marked with (§) in the application form and protocol will be published on the CPRD website in line with CPRD's transparency policy.
- I agree to inform the CPRD of the final outcome of the research study: publication, prolonged delay, completion or termination of the study.

Name: Dr Fiona M Walter Date: 29th June 2018 e-Signature (type name): Fiona M Walter

PROTOCOL INFORMATION REQUIRED

The following sections below **must** be included in the CPRD ISAC research protocol. Please refer to the guidance on '***Contents of CPRD ISAC Research Protocols***' (www.cprd.com/isac) for more information on how to complete the sections below. Pages should be numbered. All abbreviations must be defined on first use.

**Applicants must complete all sections listed below**<br>**Sections which do not apply should be completed as '*Not Applicable*'**
**A. Study Title§**<br>§*Please note: This information will be published on CPRD's website as part of its transparency policy*<br><br>The role of CA125 in the detection of ovarian cancer in symptomatic primary care patients
**B. Lay Summary (Max. 200 words)§**<br>§*Please note: This information will be published on CPRD's website as part of its transparency policy*<br><br>Ovarian cancer is the 5th most common cause of cancer related death in UK women. The majority of women are diagnosed late and only 46 out of every 100 UK women survive for 5 years after diagnosis. Early diagnosis is likely to result in better patient outcomes including survival.<br><br>However, early diagnosis is challenging. The symptoms of ovarian cancer are vague and the same symptoms occur in non-worrying medical conditions, so it is can be difficult for GPs to decide which patients need to be sent to hospital urgently for more tests and which can be reassured. Simple blood tests, such as CA125, can be used to help GPs make these decisions. However, we don't know how good CA125 is when used in primary care or what 'cut-off point' to use for an abnormal result.<br><br>In this study, we aim to determine how effective CA125 is at picking up cancer in women visiting their GP with symptoms which could be caused to ovarian cancer, and identify the most appropriate abnormal CA125 cut-off. This work will help GPs to make decisions regarding investigation and referral of symptomatic women.
**C. Technical Summary (Max. 200 words)§**<br>§*Please note: This information will be published on CPRD's website as part of its transparency policy*<br><br>Ovarian cancer has the worst prognosis of any gynaecological cancer. Early diagnosis is likely to improve survival, and, while symptoms occur in all stages, they are also common in benign conditions. Tests are needed to help distinguish malignant from benign disease in symptomatic patients.<br><br>The serum biomarker CA125 is frequently elevated in women with ovarian cancer. It is used as a first line investigation in primary care, in the UK and internationally, in patients presenting with symptoms that might be caused by ovarian cancer. Despite widespread use, the diagnostic accuracy of CA125 in the primary care population has not been established and the current 'abnormal threshold' (35u/ml) is not based on primary care data.<br><br>In this prospective cohort study, we will determine the diagnostic accuracy (sensitivity, specificity, PPV, NPV) of CA125 in a symptomatic primary care population and identify CA125 thresholds that equate to a range of risk thresholds (PPVs). As CA125 levels and ovarian cancer risk are influenced by patient variables, we will produce stratified thresholds based on key variables e.g. age. This work will allow GPs to make decisions about further investigation and referral based on patient risk.

Applicants must complete all sections listed below
Sections which do not apply should be completed as '*Not Applicable*'

D. Objectives, Specific Aims and Rationale

Study objective: To evaluate the diagnostic accuracy of CA125 in a symptomatic primary care population and develop primary care evidenced thresholds to guide further investigation and referral.

Aim 1: To determine the **overall** diagnostic accuracy of CA125 at the current threshold and identify a range of CA125 thresholds equating to different PPV's
Rationale: CA125 is advocated as the first line test in patients presenting to primary care in the UK (and several other countries) with symptoms suggestive of ovarian cancer. The National Institute of Clinical Excellence (NICE) has set a 'risk threshold' of 3% for investigation and cancer pathway referrals in symptomatic patients [1]. Research indicates that patients would opt for investigation at much lower risk thresholds [2]. We will determine the diagnostic accuracy (sensitivity, specificity, PPV, NPV) of CA125 in UK primary care at its current threshold and calculate thresholds that equate to a range of PPVs (including 1% and 3%). This will aid GPs to make informed decisions about further investigations and referrals. NICE guidance may be revised in 2019/2020, and our results would greatly facilitate guideline revision.

Aim 2: To determine the diagnostic accuracy of CA125 at the current threshold and identify a range of primary care evidenced thresholds for **patient subgroups**
Rationale: CA125 levels are affected by a number of patient variables such as age and co-morbidities. Further, some of the same factors affect the risk of ovarian cancer. Thus, the same CA125 level may equate to different PPVs in different patient subgroups e.g. <50 yrs old >50yrs old. As such, different CA125 thresholds may be required in different groups to reach the same ovarian cancer 'risk threshold'. We will determine the diagnostic performance of CA125 in specific subgroups and calculate thresholds for these subgroups which equate to a range of PPVs (including 1% and 3%). This will aid GPs to make more individualised decisions about further investigations and referrals.

Aim 3: To examine how CA125 testing, using the current threshold, impacts on the stage of diagnosis for women with values close to the current threshold
Rationale: Current guidance advocates that women with CA125 levels above 35u/ml undergo further investigation. As the test is not perfect there will be women below the threshold who have, as yet undiagnosed, ovarian cancer. It is likely that these women will go on to be diagnosed, but in the intervening period the stage of disease may have advanced. It is hoped that by diagnosing women with an elevated CA125 level earlier than might have been done without the test, that they are diagnosed at an earlier stage which may allow curative treatment and better survival. Women just above and just below the threshold have no tangible difference in their risk of ovarian cancer. However, the timeliness of diagnosis is likely to be impacted by which side of the line they fall. We will exploit this arbitrary boundary, to establish the effect of initiating investigations and subsequent follow up on the basis of a positive test result using a regression discontinuity design.

E. Study Background

Ovarian cancer has the worst prognosis of any form of gynaecological cancer and accounts for over 4000 deaths in the UK each year. Survival is stage dependant and the majority of women are not diagnosed until the disease is advanced, which contributes to the UK's poor 5-year survival rate of 46% [3, 4]. Most patients with ovarian cancer who present in primary care have common non-specific symptoms e.g. bloating. Testing in primary care can help determine which patients are at greatest risk of cancer and warrant referral, and which can be reassured.

CA125 is a high molecular weight glycoprotein of unknown function expressed by several normal human tissues. Serum CA125 levels are raised in ovarian cancer and several other malignant and benign conditions [5–7]. Levels can also vary during the menstrual cycle and in pregnancy [7]. In 2011, NICE published guidelines advocating CA125 testing in primary care patients presenting with symptoms that might represent ovarian cancer [8]. Further investigation was recommended in patients with a CA125 level above 35u/ml. Following this guidance, there was a substantial increase in CA125 testing [9]. Several other countries also advocate CA125 as an initial test for ovarian cancer in primary care [10, 11].

Despite its widespread use, the diagnostic performance of CA125 in patients presenting to primary care with symptoms suggestive of ovarian cancer has not been determined. The 'abnormal threshold' of 35u/ml, which is currently employed in both primary and secondary care throughout the world, is derived from a 1983 study in which

1% of 888 healthy patient and 82% (n=101) of patients with ovarian cancer had a CA125 level above 35u/ml [5]. CA125 has subsequently been studied extensively in secondary care, however, this research cannot readily be translated to the primary care setting as the characteristics of the population, including the incidence of ovarian cancer and other benign and malignant conditions which affect CA25 levels, is inherently different. These differences result in spectrum bias or spectrum effect, where the performance of a test varies depending on the population in which it is used [12].

In addition, use of a single CA125 threshold may not be the optimal approach in primary care, as cancer risk and CA125 levels vary between patient groups. For example, CA125 levels are significantly higher in groups of apparently healthy women under the age of 50 years than groups 50 years and over [13], while the incidence of ovarian cancer is lower in women under the age of 50 years [14]. As such, a single threshold may equate to different PPVs for ovarian cancer in different patient groups. Thresholds stratified on the basis of key variables, such as age, can be more accurate in determining disease status [15].

NICE recommends that in symptomatic patients presenting to primary care, a 3% risk (PPV) of ovarian cancer is sufficient to trigger urgent investigation or a cancer pathway referral. Patients have indicated that they would opt for investigation at lower risk thresholds e.g. 1%. In this study, we will determine what CA125 thresholds equate to different risk thresholds. The work will help guide GPs when making decisions about further investigations and referrals.

Definitions and Terminology

The term ovarian cancer can be used to encompass a number of distinct diseases which, while occurring in a similar anatomical region, differ in their tissue of origin, aetiology, molecular pathogenesis, clinical behaviour, presentation, treatment and prognosis. Ovarian cancer can be broadly divided into epithelial (>90%) and non-epithelial in origin. Non-epithelial cancers are a heterogeneous group of rare cancers that present early, have a relatively good prognosis and generally do not cause elevation in serum CA125, while epithelial cancers present late, have a poor prognosis and usually result in elevated CA125.
The term ovarian cancer is a misnomer. Epithelial ovarian cancer can arise from the epithelial lining of the ovary, fallopian tube (most common site) or the peritoneum [16]. Ovarian, fallopian tube and primary peritoneal cancers are now classified and staged using the same systems [17], and are treated collectively in current NICE guidance covering recognition and initial management.
In this protocol, the term 'ovarian cancer' will be used to describe **ovarian, fallopian tube and primary peritoneal cancer**.

F. Study Type

This is a diagnostic accuracy study in which we seek to determine the diagnostic performance of CA125 in a symptomatic primary care population. We believe this falls into the 'hypothesis testing' category as outlined in the ISAC protocol guidance.

G. Study Design

We will use a prospective cohort design to determine the diagnostic accuracy of CA125 when used in UK general practice.

H. Feasibility counts

Patients with a CA125 result:
We used the Define tool to estimate the numbers of patients with a new CA125 code and the number of patients with both a new CA125 code and a new code for ovarian cancer during our period of interest. We used Read codes for CA125 and epithelial ovarian cancer (Appendix). We applied the following restrictions-
-Date- 1st May 2011 to 1st June 2016
-Age at index date- >18 years at index date
-Gender- Female
-System- vision
-CA125 first ever code in study period
-Ovarian cancer first ever code in study period

Applicants must complete all sections listed below
Sections which do not apply should be completed as '*Not Applicable*'

This search identified **121,012 patients** with a new code for CA125 and **1423 patients** with a new code for CA125 and a new code for ovarian cancer.

Given the differences between epithelial and non-epithelial cancer (discussed in Definitions and Terminology) we will perform a sub analysis using diagnosis of epithelial ovarian cancer as the outcome. As codes for ovarian cancer within CPRD are non-specific, it is not possible to determine which CA125 tested patients have epithelial ovarian cancer on the basis of CPRD codes. As cancer registry data includes histological cancer type we will perform this subgroup analysis on patients with linked cancer registry data, which is around 57% of patients [18]. As 90% of ovarian cancer is epithelial in origin we anticipate that this analysis will include at least **68,977** CA125 tested patients of whom an estimated **730** patients will have a cancer registry documented epithelial ovarian cancer.

I. Sample size considerations

The principal objective of this study is to determine the diagnostic accuracy of CA125 when used within a symptomatic primary care population. As no study has evaluated the sensitivity and specificity of CA125 in primary care, we have used values from a large meta-analysis performed in secondary care in patients with a known pelvic mass (sensitivity 79%, specificity 78%) to perform the below calculations [19]. All calculations were performed using Stata 15.1.

With a sample consisting of 121,012 CA125 tested patients and 1423 patients with ovarian cancer, we estimate 95% confidence intervals of 76.8% to 81.1% around a sensitivity of 79%, 77.8% to 78.2% around a specificity of 78% and 3.9% to 4.3% around a PPV of 4.1%.

These confidence intervals are narrow and will allow for precision in our calculations of CA125 diagnostic accuracy for the overall population.

The second objective of this study is to determine the diagnostic accuracy of CA125 in key patient subgroups. e.g. different age groups, different ethnicities and patients with specific ovarian cancer associated symptoms. The numbers of CA125 tested women and the number of patients with ovarian cancer within each of these groups is unknown but is likely to be small for some groups. For example, we are interested in evaluating the diagnostic performance of CA125 in patients of different ethnicities as baseline levels are believed to vary between different ethnic groups [20]. In the 2011 Census of England and Wales, 3.4% of the population were classified as Black [21]. Extrapolating this to our sample, we anticipate that 4,114 of the CA125 tested women will be of Black ethnicity. This will provide 80% power to see a difference in ovarian cancer diagnoses rates between 1.7% and 2.3% in non-Black/Black women. We would also have 90% power to see a change in sensitivity from 79% to 92% in this group. Changes in either the prevalence of ovarian cancer in the tested population, the sensitivity or specificity of the test will lead to changes in the PPV. Restricting estimates of diagnostic accuracy to Black women will result in broader confidence intervals, which would be expected to be as follows if the test characteristics did not change: sensitivity (65.0-89.5%), specificity (76.7 -79.3%) and PPV (2.9-5.5%). In order to maximise our ability to evaluate test diagnostic accuracy in these subgroups we have requested data for all CA125 tested patients during our period of interest.

J. Data Linkage Required (if applicable):§

§*Please note that the data linkage/s requested in research protocols will be published by the CPRD as part of its transparency policy*

Cancer Registry- Diagnosis of ovarian cancer is the primary outcome in the study. Although concordance between CPRD and the cancer registry is high, additional cases can be identified from the registry data [22] and CPRD codes are frequently non-specific. The cancer registry contains information on cancer stage, grade and tumour size at diagnosis and histological cancer type, which will be included in the study as discussed below.

HES Admitted Patient Care (integrated data)- ethnicity is a variable in the study. Ethnicity is more frequently recorded within HES than CPRD data [23].

Office of National Statistics (ONS) Deaths Registration data- Death due to ovarian cancer will be included as a secondary outcome. This linkage is required to cross validate the cause and date of death.

Applicants must complete all sections listed below
Sections which do not apply should be completed as '*Not Applicable*'

K. Study population

The study population will consist of women who underwent a CA125 test between the 1st of May 2011 and a date 2 years before the study commences (chosen to allow 2 years of follow-up for all patients). Read codes for CA125 (listed in the appendix) will be used to define our cohort.

Inclusion criteria:
-Women ≥18 years of age at the time of the first CA125 test during the study period.
-No recorded ovarian cancer diagnosis (either within the cancer registry or CPRD data) at the time of the first CA125 test.
- ≥ 1 year of up to standard CPRD records prior to first CA125 testing.

Exclusion criteria:
-Women < 18 years of age at the time of the first CA125 test during the study period.
-Women with a recorded ovarian cancer diagnosis (either within the cancer registry or CPRD data) at the time of CA125 testing during the study period. We will not exclude patients with other comorbidities known to affect CA125 levels as we wish our study cohort to remain representative of the population in which the test is being performed.

L. Selection of comparison group(s) or controls

Internal comparison- we will compare the incidence of ovarian cancer in patients with / without an elevated CA125 level.

M. Exposures, Health Outcomes§ and Covariates
§*Please note: Summary information on health outcomes (as included on the ISAC application form above)will be published on CPRD's website as part of its transparency policy*

Primary outcome variable-
A new diagnosis of ovarian cancer recorded within the clinical record / referral files or linked cancer registry data, within 2 years of a CA125 test.
A code list for ovarian cancer (appendix), will be used to search the dataset. A follow-up period of two years from the date of initial CA125 testing has been chosen as a compromise between picking up all related cancers (which would be maximised by using a longer follow-up) and picking up unrelated cancers (which would be minimised by using a shorter follow-up). We will also stratify cancer diagnoses by the number of days the diagnosis occurred following CA125 testing.

Secondary outcome variables-
a) A new diagnosis of a cancer other than ovarian cancer, recorded within the clinical record / referral files or linked cancer registry data, within 2 years of a CA125 test. CA125 may be elevated in a number of other cancers e.g. endometrial and lung. Validated code lists for cancers developed by Professor Hamilton's group will be used.
b) Death, from 1) ovarian cancer, 2) any cancer, as recorded in CPRD data or ONS death registration data within 2 years of a CA125 test.
c) Stage of cancer as recorded in cancer registry data. It is acknowledged that this information will only be available for patients with linked cancer registry data.
d) Tumour morphology and histology, as recorded in the cancer registry. CA125 levels are known to vary by histological type [7].
e) Tumour size, as recorded in cancer registry.
f) Tumour grade as recorded in cancer registry.

Principle explanatory variable-
CA125 level.
CA125 tests will be identified from CPRD data using Read codes (appendix).

Other variables-
CA125 level and / or ovarian cancer risk are affected by a number of variables such as symptoms, comorbidities, lifestyle factors and family / patient history of cancer. We will seek to examine a number of variables including:

Applicants must complete all sections listed below
Sections which do not apply should be completed as '*Not Applicable*'

Variable	Source	Comment / rationale
**Symptoms and signs**		
Symptoms and signs within 1 month of CA125 testing	Validated code lists for symptoms related to ovarian cancer in CPRD	Risk of ovarian cancer is greater in patients presenting to primary care with certain symptoms [24]. In addition, symptoms may be related to an underlying condition other than cancer e.g. endometriosis, which may affect CA125 level.
**Patient characteristics**		
Age at time of CA125 testing	CPRD data	CA125 levels are thought to be higher in groups of younger women than older women [13]. Ovarian cancer risk is age related.
Ethnicity	CPRD data and, where available, HES data. Codes for the 16 ethnic groups recognised in the 2001 census	For analysis, these codes will be collapsed into 4 ethnic groups- 'white', 'mixed', 'Asian or Asian British', 'Chinese or other'. CA125 level is thought to vary between groups of women of different ethnicities [20].
Parity	Relevant Read codes within CPRD	CaA125 levels are thought to be lower in groups of parous vs nonporous women [25].
**Tests**		
Test results including FBC (platelet count, total white cell count, haemoglobin, platelet count), GFR, creatinine, CRP, albumin	Read codes for test results within CPRD	Some results e.g. GFR and CRP may indicate underlying conditions which affect CA125 levels. Other tests e.g. platelet count, may be predictive of ovarian cancer [26].
**Comorbidities and operations**		
Comorbidities (fibroids, endometriosis, ovarian cyst, renal failure, pre-existing cancer other than ovarian) recorded within 2 years of CA125 testing	Relevant Read codes within CPRD	Comorbidities known to affect CA125 level will be included. The risk of ovarian cancer is greater in patients with endometriosis [27].
**Personal and family history of cancer and cancer syndromes**		
Personal history of breast, endometrial, stomach, colon, small intestine, hepatobiliary, urinary tract, brain or skin cancer	Relevant Read codes within CPRD	Risk of ovarian cancer is likely to be greater in women who have had a BRCA or Lynch syndrome related cancer
Family history of breast, ovarian, endometrial, stomach, colon, small intestine, hepatobiliary, urinary tract, brain or skin cancer in first degree relatives	Relevant Read codes within CPRD	Risk of ovarian cancer may be greater in women with a family member who has had a BRCA or Lynch syndrome related cancer
BRCA mutations or Lynch syndrome	Relevant Read codes within CPRD	Risk of ovarian cancer is greater in women with a BRCA mutation or Lynch syndrome
BRCA mutations or Lynch syndrome in a first degree family member	Relevant Read codes within CPRD	Risk of ovarian cancer may be greater in women with a family member who has a BRCA mutation or Lynch syndrome

Applicants must complete all sections listed below
Sections which do not apply should be completed as '*Not Applicable*'

N. Data/ Statistical Analysis

Firstly, we will use descriptive statistics to summarise outcomes (e.g. numbers and proportions of patients diagnosed with ovarian cancer and other cancers) and variables (e.g. numbers and proportions of patients with symptom codes). All variables will be predefined prior to data analysis. An exploratory phase will assess the association between these pre-defined variables and CA125 level and predefined variables and primary outcome using regression analysis.

The principal objective of this study is to determine the diagnostic accuracy of CA125 in symptomatic primary care patients. In order to do this we will calculate the number of true positive, false positive, true negative and false negative CA125 results. From this, we will calculate the diagnostic accuracy (sensitivity, specificity, PPV and NPV) of CA125. 95% confidence intervals will be calculated for the various measures of test accuracy. This analysis will be repeated for secondary outcomes.

An important objective of the study is to determine what CA125 thresholds equate to different risk thresholds for ovarian cancer. As such, we will construct ROC curves to illustrate CA125 performance at a range of thresholds equating to various PPVs.

We expect that the diagnostic accuracy of CA125 may differ between different patient groups e.g. >50/<50 years old. We will use logistic regression to establish which factors influence the risk of having cancer, and explore whether the association between having cancer and CA125 level varies between predefined patient groups. If there is evidence of either of these associations it will suggest different test characteristics. Once the important factors have been identified, we will repeat the ROC curve analysis for these key patient subgroups.

As discussed above, CPRD codes for ovarian cancer are non-specific and will not allow us to distinguish epithelial ovarian cancer from non-epithelial types of ovarian cancer. A subgroup analysis will be performed using data for patients with linked CPRD-cancer registry data where histological cancer type can be accurately determined. We are including malignant and borderline tumours as our primary endpoint while excluding in situ lesions. Sensitivity analyses will be performed, using the morphology variable in the cancer registry, firstly excluding borderline tumours then including in situ and borderline tumours.

Our study aims to determine the performance of CA125 in symptomatic patients. However, real world use of CA125 in UK general practice may differ from intended use in symptomatic patients as set out in NICE guidelines. To test this, we will perform a sensitivity analysis using data from patients with CPRD codes for symptoms included in NICE guidance.

Finally we will perform a regression discontinuity design analysis. Regression discontinuity designs exploit arbitrary thresholds at which a certain action is taken. This has recently been used to demonstrate that the use of PSA testing in a screening population leads to an overdiagnosis of early stage prostate cancers with no change in overall mortality [28]. In this study we will use a regression discontinuity design to examine changes in early stage (TNM stages 1 and 2) and in late stage (TNM stage 3 and 4) cancers as well as overall diagnosis rates at the currently used CA125 threshold. We do not expect to see evidence of overdiagnosis and thus no change in overall diagnosis rates, combined with increases in early stage diagnoses above the threshold would support the use of CA125 testing to facilitate earlier diagnosis. Following the previous work on PSA testing we will use the user written regression discontinuity Stata module [29].

Analyses will be performed using STATA version 15.1.

O. Plan for addressing confounding

This study will assess the diagnostic accuracy of CA125 as it is used in real world UK general practice. CPRD data is largely representative of the UK general practice population.

Nevertheless, we have endeavoured to identify variables that are associated with CA125 level and ovarian cancer risk, as outlined above. We will explore the relationship between each variable, CA125 level and cancer incidence using regression analysis. Wherever possible, we will produce variable specific ROC curves.

P. Plans for addressing missing data

We will describe the extent of missing data and use appropriate approaches to evaluate its impact.

Previous studies have found that the recording of blood results within CPRD data is excellent. Linkage to cancer registry data provides us with two sources from which we can identify cancer diagnoses. As such, we anticipate having relatively complete data for our principal explanatory variable and outcomes. As with any cohort or diagnostic accuracy study, there is a risk of loss to follow-up e.g. if a patient moves away. However, we do not anticipate that the loss to follow-up rate will be significantly different between CA125 positive/negative patients prior to diagnosis.

For several of our variables and secondary outcomes we have identified more than one source of information e.g. mortality (CPRD, ONS). This will help reduce the impact of missing data.

While we anticipate having relatively complete data for our principal explanatory variable and primary outcome, we recognise that data for several other variables, e.g. personal and family history of cancer may be less complete. Any such data will be interpreted with caution and the limitations of the data will be highlighted in any related publications. Some secondary outcomes e.g. stage at diagnosis, are also likely to have some missing data. However, missing outcome data will not result in bias in estimated associations under the Missing At Random (MAR) assumption.

Q. Patient or user group involvement (if applicable)

Our group has a patient and public involvement representative, Mrs Margaret Johnson. She has given valuable input into this proposal and the protocol and will continue to be involved throughout the study.

R. Plans for disseminating and communicating study results, including the presence or absence of any restrictions on the extent and timing of publication

We aim to publish this work in peer reviewed journals. In addition, it will be presented at national and international primary care and cancer conferences. This work will form an integral part of Garth Funston's PhD thesis.
Previous routine data studies have led to the development and dissemination of paper and online risk tools [30]. We will explore the possibility of converting our findings into an e-algorithm / tool which can be used by GPs in the assessment of patients.

S. Limitations of the study design, data sources, and analytic methods

Several minor limitations exist.

One limitation is that we will be unable to conclusively identify the reasons for CA125 requests. However, we will be able to identify symptoms and signs recorded in CRPD data close to the time of CA125 testing, which may suggest a rationale for requests. As we cannot say for certain why CA125 tests were ordered, we will be unable to make firm judgements about the appropriateness of testing or whether it was conducted in line with NICE guidelines. Despite this, we will be able to demonstrate how CA125 performs as is it is currently used in UK general practice and a sensitivity analysis will be performed using data from patients with CPRD codes for symptoms included in NICE guidance.

Reliance on Read codes to identify symptoms and the presence of other variables is a limitation, as coding may be incomplete.

We have selected a 2 year follow up from the point of CA125 testing. While we believe that this is an appropriate period, it is possible that incidental ovarian cancers may occur and be diagnosed during this time or that patients with false negative CA125 results may not have re-presented and been diagnosed.

We recognise that the number of cancer patients within some of the subgroups may be limited, which in turn will limit the precision of our estimates of diagnostic accuracy for these groups. In order to maximise our ability to evaluate test diagnostic accuracy in these subgroups we have requested data for all CA125 tested patients during our period of interest.

T. References

**Applicants must complete all sections listed below**<br>**Sections which do not apply should be completed as '*Not Applicable*'**

1. National Institute of Clinical Excellence. Suspected cancer: recognition and referral. www.nice.org.uk/guidance/ng12. Accessed 26 Jul 2017.
2. Banks J, Hollinghurst S, Bigwood L, et al. Preferences for cancer investigation: A vignette-based study of primary-care attendees. Lancet Oncol. 2014;15:232–240.
3. Cancer Research UK. Ovarian cancer mortality statistics. www.cancerresearchuk.org/health-professional/cancer-statistics/statistics-by-cancer-type/ovarian-cancer/mortality. Accessed 26 Jul 2017.
4. Maringe C, Walters S, Butler J, et al. Stage at diagnosis and ovarian cancer survival: Evidence from the International Cancer Benchmarking Partnership. Gynecol Oncol. 2012;127:75–82.
5. Bast RC, Klug TL, John E St., et al. A Radioimmunoassay Using a Monoclonal Antibody to Monitor the Course of Epithelial Ovarian Cancer. N Engl J Med. 1983;309:883–887.
6. Molina R, Augé JM, Bosch X, et al. Usefulness of serum tumor markers, including progastrin-releasing peptide, in patients with lung cancer: correlation with histology. Tumour Biol. 2009;30:121–9.
7. Jacobs I, Bast RC. The CA 125 tumour-associated antigen: a review of the literature. Hum Reprod. 1989;4:1–12.
8. National Institute of Clinical Excellence. Ovarian cancer: recognition and initial management. https://www.nice.org.uk/guidance/cg122/resources/ovarian-cancer-recognition-and-initial-management-35109446543557. Accessed 14 Feb 2018.
9. Giamougiannis P, Ebef NG dit, Mahmood T. The implications of new guideline 122 On the detection of ovarian cancer. Eur J Obstet Gynecol Reprod Biol. 2016;206:e154.
10. Ireland National Cancer Control Programme. Ovarian cancer GP referral for symptomatic women. www.hse.ie/eng/services/list/5/cancer/profinfo/resources/gpreferrals/Ovarian-Cancer-Referral-Guidelines.pdf. Accessed 2 Dec 2017.
11. Cancer Australia. Assessment of symptoms that may be ovarian cancer: a guide for GPs. In: 2015. https://canceraustralia.gov.au/publications-and-resources/cancer-australia-publications/assessment-symptoms-may-be-ovarian-cancer-guide-gps. Accessed 14 Feb 2018.
12. Usher-Smith JA, Sharp SJ, Griffin SJ. The spectrum effect in tests for risk prediction, screening, and diagnosis. BMJ. doi: 10.1136/bmj.i3139
13. Haga Y, Sakamoto K, Egami H, Yoshimura R, Akagi M. Evaluation of serum CA125 values in healthy individuals and pregnant women. Am J Med Sci. 1986;292:25–9.
14. Cancer Research UK. Ovarian cancer incidence statistics. www.cancerresearchuk.org/health-professional/cancer-statistics/statistics-by-cancer-type/ovarian-cancer/incidence#heading-One. Accessed 14 Feb 2018.
15. Quinn MM, Kao CN, Ahmad AK, et al. Age-stratified thresholds of anti-Müllerian hormone improve prediction of polycystic ovary syndrome over a population-based threshold. Clin Endocrinol (Oxf). 2017;87:733–740.
16. Karnezis AN, Cho KR, Gilks CB, Pearce CL, Huntsman DG. The disparate origins of ovarian cancers: Pathogenesis and prevention strategies. Nat Rev Cancer. 2017;17:65–74.
17. Meinhold-Heerlein I, Fotopoulou C, Harter P, et al. The new WHO classification of ovarian, fallopian tube, and primary peritoneal cancer and its clinical implications. Arch Gynecol Obstet. 2016;293:695–700.

Applicants must complete all sections listed below
Sections which do not apply should be completed as '*Not Applicable*'

18. Clinical Practice Research Datalink. CPRD linked data. https://www.cprd.com/dataAccess/linkeddata.asp. Accessed 25 Apr 2018.
19. Ferraro S, Braga F, Lanzoni M, et al. Serum human epididymis protein 4 vs. carbohydrate antigen 125 for ovarian cancer diagnosis: A systematic review. Biochim Clin. 2013;37:179–189.
20. Johnson CC, Kessel B, Riley TL, et al. The epidemiology of CA-125 in women without evidence of ovarian cancer in the Prostate, Lung, Colorectal and Ovarian Cancer (PLCO) Screening Trial. Gynecol Oncol. 2008;110:383–389.
21. Gov.UK. Ethnicity facts and figures. https://www.ethnicity-facts-figures.service.gov.uk/ethnicity-in-the-uk/population-by-ethnicity. Accessed 25 Apr 2018.
22. Boggon R, Van Staa TP, Chapman M, et al. Cancer recording and mortality in the General Practice Research Database and linked cancer registries. Pharmacoepidemiol Drug Saf. 2013;22:168–175.
23. Mathur R, Bhaskaran K, Chaturvedi N, et al. Completeness and usability of ethnicity data in UK-based primary care and hospital databases. J Public Health (Oxf). 2014;36:684–692.
24. Hamilton W, Peters TJ, Bankhead C, Sharp D. Risk of ovarian cancer in women with symptoms in primary care: population based case-control study. BMJ. 2009;339:b2998.
25. Lowe KA, Shah C, Wallace E, et al. Effects of personal characteristics on serum CA125, mesothelin, and HE4 levels in healthy postmenopausal women at high-risk for ovarian cancer. Cancer Epidemiol Biomarkers Prev. 2008;17:2480–2487.
26. Hippisley-Cox J, Coupland C. Identifying women with suspected ovarian cancer in primary care: derivation and validation of algorithm. BMJ. 2011;344:d8009–d8009.
27. Kvaskoff M, Mu F, Terry KL, et al. Endometriosis: A high-risk population for major chronic diseases? Hum Reprod Update. 2014;21:500–516.
28. Jonathan Shoag, Joshua Halpern, Brian Eisner et al. Efficacy of Prostate-Specific Antigen Screening: Use of Regression Discontinuity in the PLCO Cancer Screening Trial. JAMMA Oncol. 2015;1:984–986.
29. Nichols A. RD: Stata module for regression discontinuity estimation. https://ideas.repec.org/c/boc/bocode/s456888.html. Accessed 27 Jun 2018.
30. Hamilton W, Green T, Martins T, et al. Evaluation of risk assessment tools for suspected cancer in general practice: A cohort study. Br J Gen Pract. doi: 10.3399/bjgp13X660751

List of Appendices *(Submit all appendices as separate documents to this application)*

Appendix 1: CA125 codes
Appendix 2: Ovarian cancer codes

Applicants must complete all sections listed below<br>Sections which do not apply should be completed as '*Not Applicable*'
