# Supplementary material for: The diagnostic performance of CA125 for the detection of ovarian and non-ovarian cancer in primary care: A population-based cohort study
Source: PLoS Med. 2020 Oct 28;17(10):e1003295. doi: 10.1371/journal.pmed.1003295 (PMC7592785; doi:10.1371/journal.pmed.1003295)
Supplement: S2 Text — ISAC, Independent Scientific Advisory Committee. (PDF) [file pmed.1003295.s002.pdf]

Dear ISAC Secretariat,

RE: Protocol 18_184: "The role of CA125 in the detection of ovarian cancer in symptomatic primary care patients"

We would like to request the following minor amendments to the above approved protocol:

1) <u>Amendment 1</u>: Rather than accepting a code for ovarian cancer in CPRD or in NCRAS we will only accept an ovarian cancer record in NCRAS data. This will necessitate only using patients with NCRAS linkage for this aspect of the study. There are 2 reasons for this change:

    -Concordance between CPRD and NCRAS data is variable with regards to cancer recording [1]. A paper, published after our ISAC submission, highlights that solely relying on CPRD for cancer case identification could affect the results of diagnostic accuracy studies, such as ours, due to the potential for misclassification bias [1]. While compiling a list of Read codes for this study we found a number of nonspecific codes which may or may not indicate the presence of ovarian cancer- this could lead to misclassification bias. NCRAS reports a near 100% case ascertainment, collects data from multiple sources and is generally considered the gold standard for cancer recording in population cancer research and so we feel it is appropriate to use NCRAS to identify cancer cases in our study.

    -As noted in the protocol, we wish to perform a sensitivity analysis excluding borderline ovarian tumours- this is not possible using CPRD cancer codes due to their non-specific nature (morphology and topographical NCRAS codes are needed).

The sample should still be adequate to determine diagnostic accuracy of CA125 with a high degree of precision: 72,182 patients have linkage in the dataset and, based on our feasibility count, we would assume that up to 849 women to have ovarian cancer. Using estimated sensitivity (79%) and specificity (78%) from the sample size considerations section of our protocol, we would anticipate narrow confidence intervals around measures of diagnostic accuracy (particularly our main measure which is PPV): PPV 4.1% (95% CI: 3.8-4.4), NPV 99.7% (95% CI:99.6-99.7), sensitivity 79% (95% CI: 76.1-81.7), specificity 78% (95% CI: 77.7-78.3). These are very similar to the CI estimates in the approved ISAC protocol.

2) <u>Amendment 2</u>: We will use a 1 year rather than a 2 year follow-up period post CA125 testing. While some studies use a 2 year period the majority of similar recent studies (several of which have been published since we submitted our original ISAC protocol) have used a 1 year period [2-3]. We believe that a 1 year period will be sufficient for the majority of ovarian cancers to be diagnosed following CA125 testing and will minimise the number of incidental ovarian cancers included in our analysis.

3) <u>Amendment 3</u>: In the protocol we state that we will exclude patients without 1 year of up-to-standard follow-up prior to CA125 testing. We wish to change this to exclude patients whose practices are not up-to-standard at the point of CA125 testing (the focus of our study is activity post CA125 testing rather than prior to CA125 testing).

4) <u>Amendment 4:</u> We wish to add the following exclusion criteria- "Patients with a CA125 record prior to the study start date but within 12 months of the initial CA125 test during the study period". This is because evidence has emerged since we submitted the ISAC protocol that the PPV of a repeat test may be different to that of an initial test [2].

These changes do not fundamentally alter the design or aim of the study and appear to fall under the minor amendment category in your document "Guidance on Resubmissions and Amendments of ISAC Research Protocols". A protocol (with these amendments) will be submitted with the paper when we come to publish our results.

Please do not hesitate to contact me if you require further information.

Best wishes,

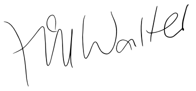

Fiona Walter

Study Chief Investigator

References
[1] Badrick E, Renehan I, Renehan AG. Linkage of the UK Clinical Practice Research Datalink with the national cancer registry. Eur J Epidemiol. 2019;34:101-102.
[2] Watson J, Salisbury C, Banks J, Whiting P, Hamilton W. Predictive value of inflammatory markers for cancer diagnosis in primary care: a prospective cohort study using electronic health records. BJC. 2019;120:1045–1051.
[3] Watson J, Jones, H, Banks J, Whiting P, Salisbury C, Hamilton W. Use of multiple inflammatory marker tests in primary care: using Clinical Practice Research Datalink to evaluate accuracy**.** 2019;69:e462-e469.
