# Supplementary material for: The diagnostic performance of CA125 for the detection of ovarian and non-ovarian cancer in primary care: A population-based cohort study
Source: PLoS Med. 2020 Oct 28;17(10):e1003295. doi: 10.1371/journal.pmed.1003295 (PMC7592785; doi:10.1371/journal.pmed.1003295)
Supplement: S3 Text — STARD, Standards for Reporting of Diagnostic Accuracy Studies. (PDF) [file pmed.1003295.s003.pdf]

Section & Topic	No	Item	Reported in
**TITLE OR ABSTRACT**			
	**1**	Identification as a study of diagnostic accuracy using at least one measure of accuracy (such as sensitivity, specificity, predictive values, or AUC)	Abstract, para 2
**ABSTRACT**			
	**2**	Structured summary of study design, methods, results, and conclusions (for specific guidance, see STARD for Abstracts)	Abstract
**INTRODUCTION**			
	**3**	Scientific and clinical background, including the intended use and clinical role of the index test	Introduction, para 1-4
	**4**	Study objectives and hypotheses	Introduction, para 5
**METHODS**			
Study design	**5**	Whether data collection was planned before the index test and reference standard were performed (prospective study) or after (retrospective study)	Methods, para 2
Participants	**6**	Eligibility criteria	Methods, para 4-5
	**7**	On what basis potentially eligible participants were identified (such as symptoms, results from previous tests, inclusion in registry)	Methods, para 4-5
	**8**	Where and when potentially eligible participants were identified (setting, location and dates)	Methods, para 2 & 4-5
	**9**	Whether participants formed a consecutive, random or convenience series	Methods, para 4-5
Test methods	**10a**	Index test, in sufficient detail to allow replication	Methods, para 4-5
	**10b**	Reference standard, in sufficient detail to allow replication	Methods, para 2 & para 6
	**11**	Rationale for choosing the reference standard (if alternatives exist)	Methods, para 2 & para 6
	**12a**	Definition of and rationale for test positivity cut-offs or result categories of the index test, distinguishing pre-specified from exploratory	Methods, para 5 & 12
	**12b**	Definition of and rationale for test positivity cut-offs or result categories of the reference standard, distinguishing pre-specified from exploratory	Methods, para 6
	**13a**	Whether clinical information and reference standard results were available to the performers/readers of the index test	Methods, para 4-5
	**13b**	Whether clinical information and index test results were available to the assessors of the reference standard	Methods, para 6
Analysis	**14**	Methods for estimating or comparing measures of diagnostic accuracy	Methods, para 12-15
	**15**	How indeterminate index test or reference standard results were handled	Methods, para 4-6
	**16**	How missing data on the index test and reference standard were handled	Methods, para 4-5 & figure 1
	**17**	Any analyses of variability in diagnostic accuracy, distinguishing pre-specified from exploratory	Methods, para 14
	**18**	Intended sample size and how it was determined	S1 Protocol and S1 Protocol Amendment
**RESULTS**			
Participants	**19**	Flow of participants, using a diagram	Figure 1
	**20**	Baseline demographic and clinical characteristics of participants	Results, para 1-2 & 5 & table 2
	**21a**	Distribution of severity of disease in those with the target condition	Results, para 3
	**21b**	Distribution of alternative diagnoses in those without the target condition	Results, table 1 & table 4
	**22**	Time interval and any clinical interventions between index test and reference standard	Results, para 2
Test results	**23**	Cross tabulation of the index test results (or their distribution) by the results of the reference standard	Results, Table 3
	**24**	Estimates of diagnostic accuracy and their precision (such as 95% confidence intervals)	Results, Table 3
	**25**	Any adverse events from performing the index test or the reference standard	N/A
**DISCUSSION**			

	**26**	Study limitations, including sources of potential bias, statistical uncertainty, and generalisability	Discussion, para 2-5
	**27**	Implications for practice, including the intended use and clinical role of the index test	Discussion, para 9-14
**OTHER INFORMATION**			
	**28**	Registration number and name of registry	N/A
	**29**	Where the full study protocol can be accessed	Appendix 1 and 2
	**30**	Sources of funding and other support; role of funders	Funding section, acknowledgements section
