# Supplementary material for: The diagnostic performance of CA125 for the detection of ovarian and non-ovarian cancer in primary care: A population-based cohort study
Source: PLoS Med. 2020 Oct 28;17(10):e1003295. doi: 10.1371/journal.pmed.1003295 (PMC7592785; doi:10.1371/journal.pmed.1003295)
Supplement: S4 Text — (PDF) [file pmed.1003295.s004.pdf]

S4 Text. Logistic regression model specifications.

The Odds ratios for terms included in each model and the location of knots in each model are described in this appendix in order to aid interpretation and enable study replication. In all models, log CA125 was centred on a value of 3 prior to analysis. Where age was included, it was centred on a value of 55.

Table Ai. Knots included in the overall CA125 ovarian cancer model (women of all ages). Results displayed in Figure 2.

**Knot**	**Placement on centred log CA125, (Quantile)**
k1	-1.390562 (0.05)
k2	-0.8027754 (0.275)
k3	-0.5150933 (0.5)
k4	-0.1667867 (0.725)
k5	0.7376696 (0.95)

Table Aii. Odds ratios for terms included in the overall CA125 ovarian cancer model (women of all ages). Results displayed in Figure 2.

**Variable**	**OR (95% CI)**
CA125 spline 1	1.93881 (0.1789538 - 21.00534)
CA125 spline 2	0.0105733 (6.00×10^{-09} - 18640.92)
CA125 spline 3	3.08×10^{27} (0.0000101 - 9.42×10^{59})
CA125 spline 4	7.66×10^{-50} (1.03×10^{-89} - 5.72×10^{-10})
Constant	*0.0018886 (0.0001076 - 0.033156)*

Table Bi. Knots included in the overall CA125 all cancer model (women of all ages). Results displayed in Figure 2.

Knot	**Placement on centred log CA125, (Quantile)**
k1	-1.208241 (0.10)
k2	-0.5150933 (0.5)
k3	0.3322045 (0.90)

Table Bii. Odds ratios for terms included in the overall CA125 all cancer model (women of all ages). Results displayed in Figure 2.

**Variable**	**OR (95% CI)**
CA125 spline 1	1.506687 (1.205222 - 1.883559)
CA125 spline 2	1.774489 (1.472288 - 2.138719)
Constant	0.0239013 (0.0201886 - 0.0282968)

Table Ci. Knots included in the overall CA125 invasive ovarian cancer model (women of all ages). Results displayed in Figure 2.

**Knot**	**Placement on centred log CA125, (Quantile)**
k1	-1.390562 (0.05)
k2	-0.8027754 (0.275)
k3	-0.5150933 (0.5)
k4	-0.1667867 (0.725)
k5	0.7376696 (0.95)

Table Cii. Odds ratios for terms included in the overall CA125 invasive ovarian cancer model (women of all ages). Results displayed in Figure 2.

**Variable**	**OR (95% CI)**
CA125 spline 1	0.918744 (0.085896 - 9.826892)
CA125 spline 2	0.0015715 (8.94×10^{-11} - 27620.28)
CA125 spline 3	2.05×10^{40} (0.9907357 - 4.23×10^{80})
CA125 spline 4	1.74×10^{-72} (4.8×10^{-124} - 6.36×10^{-21})
Constant	*0.0005503*

Table Di. Knots included in the <50 years CA125 ovarian cancer model. Results displayed in S4 Fig.

**Knot**	**Placement on centred log CA125, (Quantile)**
k1	-1.295252 (0.05)
k2	-0.6021047 (0.35)
k3	-0.1667867 (0.65)
k4	0.7358818 (0.95)

Table Dii. Odds ratios for terms included in the <50 years CA125 ovarian cancer model. Results displayed in S4 Fig.

**Variable**	**OR (95% CI)**
CA125 spline 1	0.7911065 (0.0342347 - 18.28112)
CA125 spline 2	801.5433 (0.2558281 - 2511342)
CA125 spline 3	1.37×10^{-10} (1.86×10^{-21} - 10.16702)
Constant	*0.0003367 (0.0000169 - 0.0066908)*

Table Ei. Knots included in the ≥50 years CA125 ovarian cancer model. Results displayed in S4 Fig.

**Knot**	**Placement on centred log CA125, (Quantile)**
k1	-1.410765 (0.05)
k2	-0.9205585 (0.275)
k3	-0.6021047 (0.5)
k4	-0.2274113 (0.725)
k5	0.7376696 (0.95)

Table Eii. Odds ratios for terms included in the ≥50 years CA125 ovarian cancer model. Results displayed in S4 Fig.

**Variable**	**OR (95% CI)**
CA125 spline 1	1.930133 (0.1115593 - 33.39403)
CA125 spline 2	0.0000447 4.05×10^{-15} - 492193.3)
CA125 spline 3	3.49×10^{34} (0.000021 - 5.80×10^{73})
CA125 spline 4	3.43×10^{-57} (1.2×10^{-100} - 9.73×10^{-14})
Constant	0.0023171 (0.0000615 - 0.0872512)

Table Fi. Knots included in the <50 years CA125 all cancer model. Results displayed in S4 Fig.

**Knot**	**Placement on centred log CA125, (Quantile)**
k1	-1.05409 (0.1)
k2	-0.4350506 (0.5)
k3	0.4226314 (0.9)

Table Fii. Odds ratios for terms included in the <50 years CA125 all cancer model. Results displayed in S4 Fig.

**Variable**	**OR (95% CI)**
CA125 spline 1	1.549178 (0.8615406 - 2.785653)
CA125 spline 2	1.791592 (1.057134 - 3.036324)
Constant	*0.0088448 (0.0060494 - 0.012932)*

Table Gi. Knots included in the ≥50 years CA125 all cancer model. Results displayed in S4 Fig.

**Knot**	**Placement on centred log CA125, (Quantile)**
k1	-1.410765 (0.05)
k2	-0.9205585 (0.275)
k3	-0.6021047 (0.5)
k4	-0.2274113 (0.725)
k5	0.7376696 (0.95)

Table Gii. Odds ratios for terms included in the ≥50 years CA125 all cancer model. Results displayed in S4 Fig.

**Variable**	**OR (95% CI)**
CA125 spline 1	2.562947 (1.278854 - 5.136395)
CA125 spline 2	0.0031644 (0.000011 - 0.9122155)
CA125 spline 3	2.35×10^{14} (32466.81 - 1.70×10^{24})
CA125 spline 4	7.58×10^{-21} (3.87×10^{-32} - 1.48×10^{-09})
Constant	*0.0640786 (0.0268515 - 0.1529178)*

Table Hi. Knots included in the <50 years CA125 invasive ovarian cancer model. Results displayed in S4 Fig.

**Knot**	**Placement on centred log CA125, (Quantile)**
k1	-1.05409 (0.1)
k2	-0.4350506 (0.5)
k3	0.4226314 (0.9)

Table Hii. Odds ratios for terms included in the <50 years CA125 invasive ovarian cancer model. Results displayed in S4 Fig.

Variable	OR (95% CI)
CA125 spline 1	9.660362 (0.4816256 - 193.7659)
CA125 spline 2	0.5386086 (0.0457517 - 6.340725)
Constant	*0.0018771 (0.0004428 - 0.0079571)*

Table Ii. Knots included in the ≥50 years CA125 invasive ovarian cancer model. Results displayed in S4 Fig.

Knot	Placement on centred log CA125, (Quantile)
k1	-1.410765 (0.05)
k2	-0.9205585 (0.275)
k3	-0.6021047 (0.5)
k4	-0.2274113 (0.725)
k5	0.7376696 (0.95)

Table Iii. Odds ratios for terms included in the ≥50 years CA125 invasive ovarian cancer model. Results displayed in S4 Fig.

Variable	OR (95% CI)
CA125 spline 1	0.9148951 (.0630414 - 13.27752)
CA125 spline 2	2.92×10^{-07} (1.90×10^{-18} - 44696.24)
CA125 spline 3	8.04×10^{51} (232525 - 2.78×10^{98})
CA125 spline 4	7.54×10^{-85} (4.1×10^{-139} - 1.38×10^{-30})
Constant	0.000716 (0.000019 - 0.0269587

Table Ji. Knots included in the age / CA125 ovarian cancer model. Results displayed in Figure 3.

Knot	Placement on centred age and centred log CA125, (Quantile)
Age k1	-24 (0.05)
Age k2	-9 (0.275)
Age k3	-1 (0.5)
Age k4	10 (0.725)
Age k5	27 (0.95)
CA125 k1	-1.390562 (0.05)
CA125 k2	-0.8027754 (0.275)
CA125 k3	-0.5150933 (0.5)
CA125 k4	-0.1667867 (0.725)
CA125 k5	0.7376696 (0.95)

Table Jii. Odds ratios for terms included in the age / CA125 - ovarian cancer model. Results displayed in Figure 3.

Variable	OR (95% CI)
Age spline 1	0.9263145 (0.8896194 - 0.9645232)
Age spline 2	1.746288 (1.364622 - 2.234702)
Age spline 3	0.1047997 (0.0284088 - 0.3866058)

Age spline 4	7.06957 (1.294418 - 38.61104)
CA125 spline 1	2.26625 (0.2010149 - 25.54979)
CA125 spline 2	0.0046567 ($2.28x10^{-09}$ - 9515.724)
CA125 spline 3	$1.25x10^{31}$ (0.0223821 - $6.99x10^{63}$)
CA125 spline 4	$3.40x10^{-56}$ ($2.33x10^{-96}$ - $4.96x10^{-16}$)
Constant	*0.0002362 (0.0000114 - 0.0048961)*

Table Ki. Knots included in the age / CA125 - all cancer model. Results displayed in S6 Fig.

**Knot**	**Placement on centred age and centred log CA125, (Quantile)**
Age k1	-24 (0.05)
Age k2	-9 (0.275)
Age k3	-1 (0.5)
Age k4	10 (0.725)
Age k5	27 (0.95)
CA125 k1	-1.390562 (0.05)
CA125 k2	-0.8027754 (0.275)
CA125 k3	-0.5150933 (0.5)
CA125 k4	-0.1667867 (0.725)
CA125 k5	0.7376696 (0.95)

Table Kii. Odds ratios for terms included in the age / CA125 - all cancer model. Results displayed in S6 Fig.

**Variable**	**OR (95% CI)**
Age spline 1	0.9763361 (0.9505021 - 1.002872)
Age spline 2	1.475666 (1.280135 - 1.701064)
Age spline 3	0.2244424 (0.1107301 - 0.4549296)
Age spline 4	3.271308 (1.359359 - 7.872425)
CA125 spline 1	2.378991 (1.326446 - 4.266736)
CA125 spline 2	0.0457364 (0.0011464 - 1.824658)
CA125 spline 3	$4.92x10^{11}$ (819.8629 - $2.96x10^{20}$)
CA125 spline 4	$9.51x10^{-19}$ ($4.97x10^{-30}$ - $1.82x10^{-07}$)
Constant	*0.006743 (0.0028158 - 0.0161475)*

Table Li. Knots included in the age / CA125 – invasive ovarian cancer model. Results displayed in S5 Fig.

**Knot**	**Placement on centred age and centred log CA125, (Quantile)**
Age k1	-24 (0.05)
Age k2	-9 (0.275)
Age k3	-1 (0.5)
Age k4	10 (0.725)
Age k5	27 (0.95)
CA125 k1	-1.390562 (0.05)
CA125 k2	-0.8027754 (0.275)
CA125 k3	-0.5150933 (0.5)

CA125 k4	-0.1667867 (0.725)
CA125 k5	0.7376696 (0.95)

Table Lii. Odds ratios for terms included in the age / CA125 – invasive ovarian cancer model. Results displayed in S5 Fig.

**Variable**	**OR (95% CI)**
Age spline 1	0.9415075 (0.8856343 - 1.000906)
Age spline 2	1.829243 (1.307877 - 2.558445)
Age spline 3	0.0776767 (0.014373 - 0.4197926)
Age spline 4	10.23064 (1.236766 - 84.62885)
CA125 spline 1	1.069068 (0.0959747 - 11.90841)
CA125 spline 2	0.0007832 (3.80×10^{-11} - 16157.52)
CA125 spline 3	8.33×10^{43} (1958.054 - 3.54×10^{84})
CA125 spline 4	3.36×10^{-79} (3.8×10^{-131} - 2.98×10^{-27})
Constant	*0.0000571 (2.07×10^{-06} - 0.0015762)*
