# Supplementary material for: The diagnostic performance of CA125 for the detection of ovarian and non-ovarian cancer in primary care: A population-based cohort study
Source: PLoS Med. 2020 Oct 28;17(10):e1003295. doi: 10.1371/journal.pmed.1003295 (PMC7592785; doi:10.1371/journal.pmed.1003295)
Supplement: S1 Table — (PDF) [file pmed.1003295.s005.pdf]

S1 Table. Read codes and terms used to identify CA125 tested women.

Medcode	Read code	Read term
14565	44a6.00	CA125 level
108230	44a6000	Serum CA 125 (cancer antigen 125) level
9228	44a1.00	Carbohydrate antigen 125 level
