# Supplementary material for: The diagnostic performance of CA125 for the detection of ovarian and non-ovarian cancer in primary care: A population-based cohort study
Source: PLoS Med. 2020 Oct 28;17(10):e1003295. doi: 10.1371/journal.pmed.1003295 (PMC7592785; doi:10.1371/journal.pmed.1003295)
Supplement: S2 Table — (PDF) [file pmed.1003295.s006.pdf]

S2 Table. Ovarian cancer by stage of diagnosis.

Stage	N (%)
I	138 (30.3)
II	34 (7.5)
III	142 (31.1)
IV	67 (14.7)
Missing	75 (16.4)
