# Supplementary material for: The diagnostic performance of CA125 for the detection of ovarian and non-ovarian cancer in primary care: A population-based cohort study
Source: PLoS Med. 2020 Oct 28;17(10):e1003295. doi: 10.1371/journal.pmed.1003295 (PMC7592785; doi:10.1371/journal.pmed.1003295)
Supplement: S3 Table — (PDF) [file pmed.1003295.s007.pdf]

S3 Table. Behaviour and histology of ovarian tumours by age group (<50 years and ≥50 years).

**Behaviour and Histology**	**<50 years**	**≥50 years**	**All ages**
**Invasive**			
Epithelial:			
Serous	17	157	174
Mucinous	6	16	22
Clear Cell	0	17	17
Endometrioid	4	16	20
Other	2	15	17
Unknown	4	69	73
Non-epithelial	5	8	13
Unknown	2	20	22
**Borderline**			
Borderline	40	58	98
Total	***80***	***376***	***456***
