# Supplementary material for: The diagnostic performance of CA125 for the detection of ovarian and non-ovarian cancer in primary care: A population-based cohort study
Source: PLoS Med. 2020 Oct 28;17(10):e1003295. doi: 10.1371/journal.pmed.1003295 (PMC7592785; doi:10.1371/journal.pmed.1003295)
Supplement: S4 Table — (PDF) [file pmed.1003295.s008.pdf]

S4 Table. Frequencies of non-ovarian cancers included in the 'other' group in table 4.

**Cancer type**<br>**(ICD10 codes)**	**N**
In situ: Cervix<br>(D06)	47
Melanoma<br>(C43)	21
In situ: Breast<br>(D05)	23
In situ: Other and unspecified<br>(D09)	18
Cervix<br>(C53)	18
Endocrine<br>(C37,C73,C75,D44)	13
CNS<br>(C71,C72,D43)	11
In situ: Melanoma<br>(D03)	9
Other and ill-defined digestive organs<br>(C26)	8
Mesothelioma<br>(C45)	8
In situ: Other and unspecified digestive organs<br>(D01)	7
Female genital organs: other<br>(C57.8,C57.9)	7
Secondary: other<br>(C79)	6
Connective and soft tissue<br>(C49)	4
Oropharyngeal<br>(C02, C04, C06, C09)	4
In situ: Other and unspecified genital organ<br>(D07)	4
Vagina<br>(C52)	3

Secondary and unspecified: lymph nodes<br>(C77)	3
Vulva<br>(C51)	2
Neoplasm of uncertain behaviour: other<br>(D48)	2
Retroperitoneum<br>(C48.0)	1
Neoplasm of uncertain behaviour: digestive organ<br>(D37.9)	1
Neoplasm of uncertain behaviour: placenta<br>(D39.2)	1
Nasal<br>(C30)	1
Bone<br>(C40)	1
Other and Ill-defined sites<br>(76)	1
