# Supplementary figures and images for: The diagnostic performance of CA125 for the detection of ovarian and non-ovarian cancer in primary care: A population-based cohort study
Source: PLoS Med. 2020 Oct 28;17(10):e1003295. doi: 10.1371/journal.pmed.1003295 (PMC7592785; doi:10.1371/journal.pmed.1003295)

S1 Fig. Estimated probabilities of ovarian cancer at an extended range of CA125 levels.

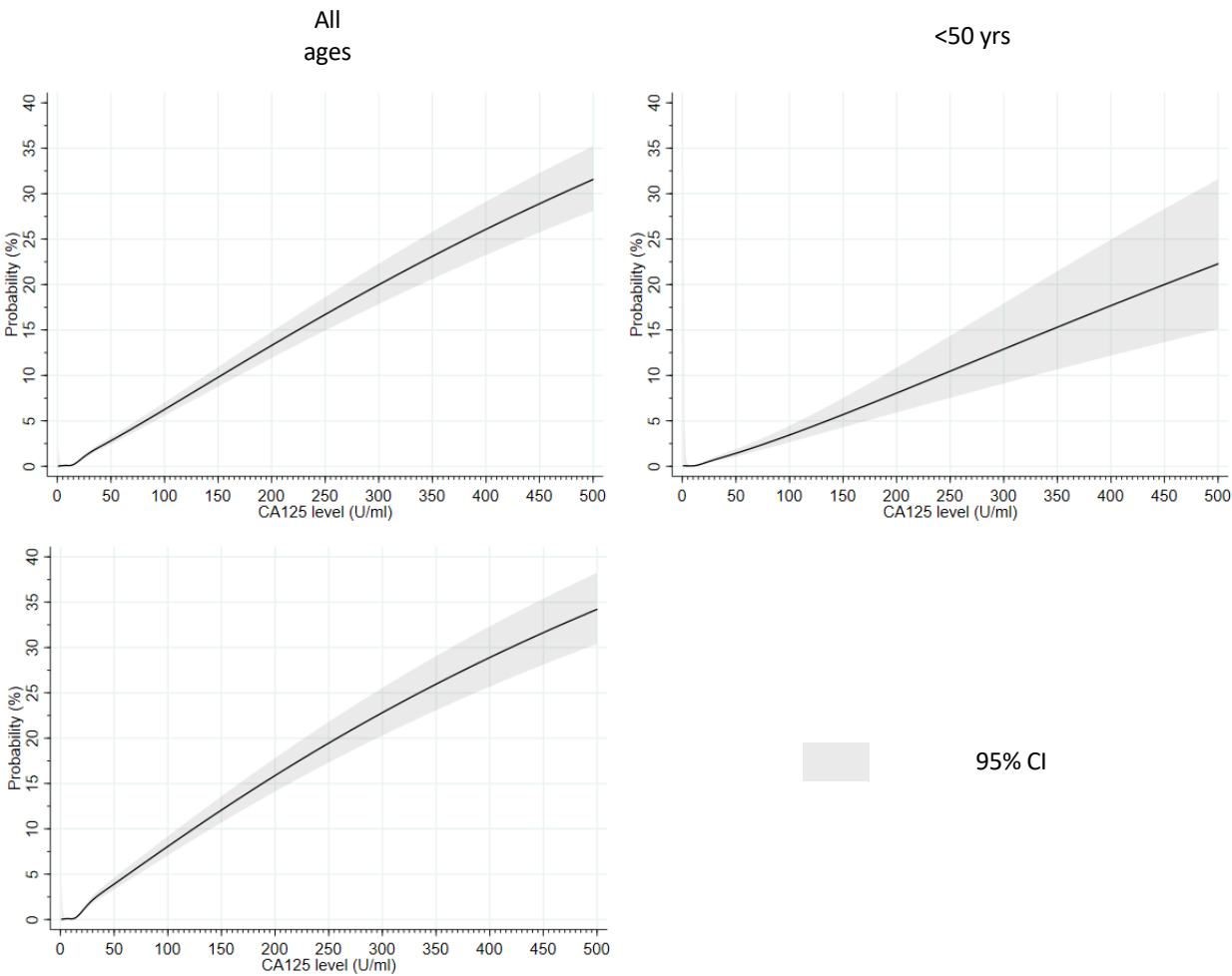

Supplement: S1 Fig — (PDF) [file pmed.1003295.s009.pdf]

S2 Fig. Estimated probabilities of invasive ovarian cancer at an extended range of CA125 levels.

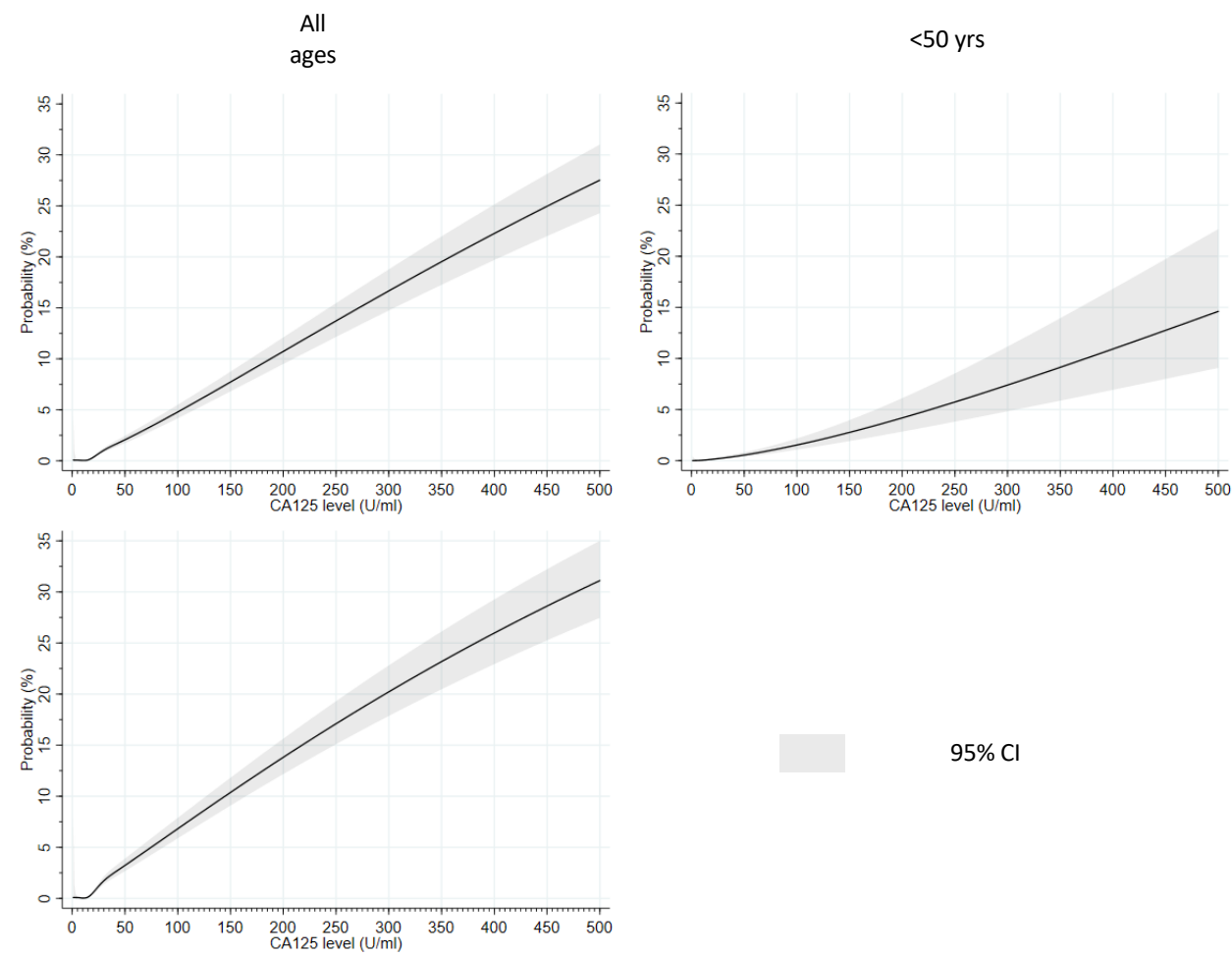

Supplement: S2 Fig — (PDF) [file pmed.1003295.s010.pdf]

S3 Fig. Estimated probabilities of all cancer at an extended range of CA125 levels.

All  
ages

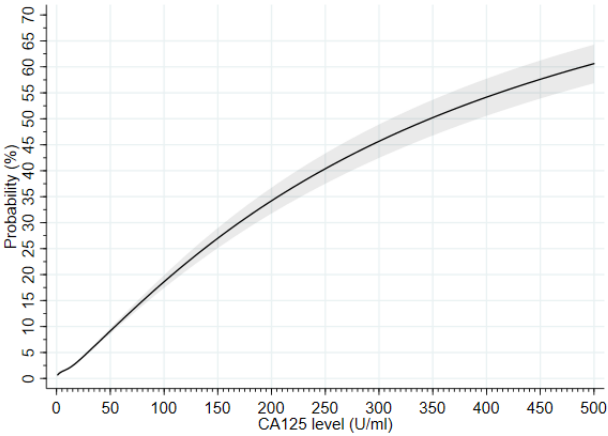

<50 yrs

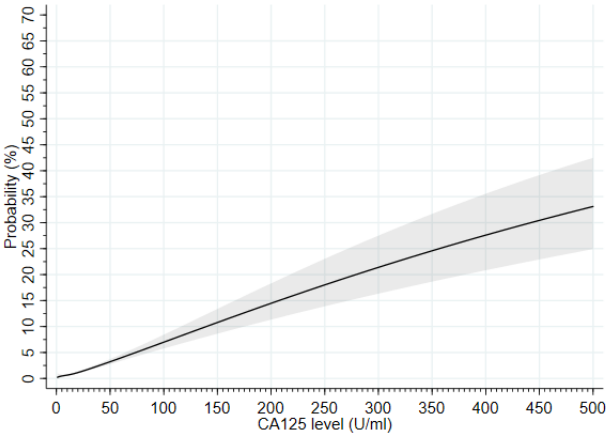

≥50 yrs

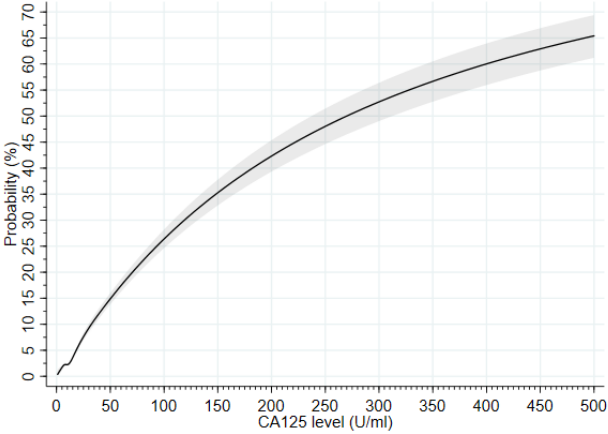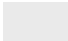

95% CI

Supplement: S3 Fig — (PDF) [file pmed.1003295.s011.pdf]
