# Supplementary material for: The diagnostic performance of CA125 for the detection of ovarian and non-ovarian cancer in primary care: A population-based cohort study
Source: PLoS Med. 2020 Oct 28;17(10):e1003295. doi: 10.1371/journal.pmed.1003295 (PMC7592785; doi:10.1371/journal.pmed.1003295)
Supplement: S4 Fig — Estimated probabilities up to 8% for each cancer type in women <50 and ≥50 years of age are shown, save for invasive cancer in the <50 years group, in which a CA125 of 317 U/ml was required to reach an 8% probability. (PDF) [file pmed.1003295.s012.pdf]

S4 Fig. Relationship between CA125 level and estimated probability of ovarian cancer, invasive ovarian cancer and all cancers in women <50 years and ≥50 years of age

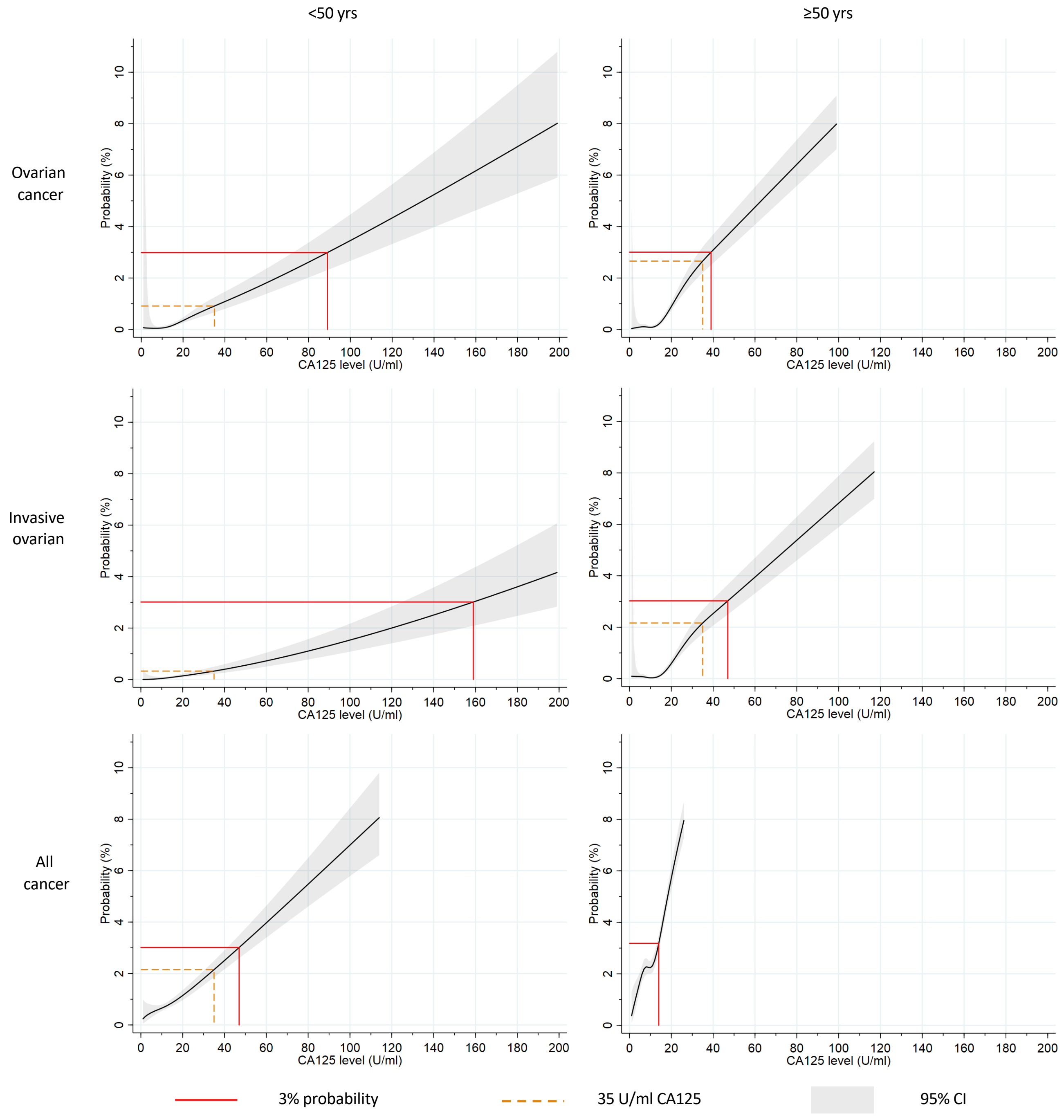

Estimated probabilities up to 8% for each cancer type in women <50 and ≥50 years of age are shown, save for invasive cancer in the <50 years group where a CA125 of 317 U/ml was required to reach an 8% probability.
