# Supplementary material for: The diagnostic performance of CA125 for the detection of ovarian and non-ovarian cancer in primary care: A population-based cohort study
Source: PLoS Med. 2020 Oct 28;17(10):e1003295. doi: 10.1371/journal.pmed.1003295 (PMC7592785; doi:10.1371/journal.pmed.1003295)
Supplement: S5 Fig — Probabilities are shown in relation to CA125 level for women of 30, 40, 50, 60, 70, and 80 years of age. CA125 levels that correspond to the closest integer probabilities of 3% are indicated in red (not displayed in 40 years of age- reached at 191 U/ml). The 95% confidence intervals are displayed. (PDF) [file pmed.1003295.s013.pdf]

S5 Fig. Relationship between CA125 level and estimated probability of invasive ovarian cancer for women of different ages.

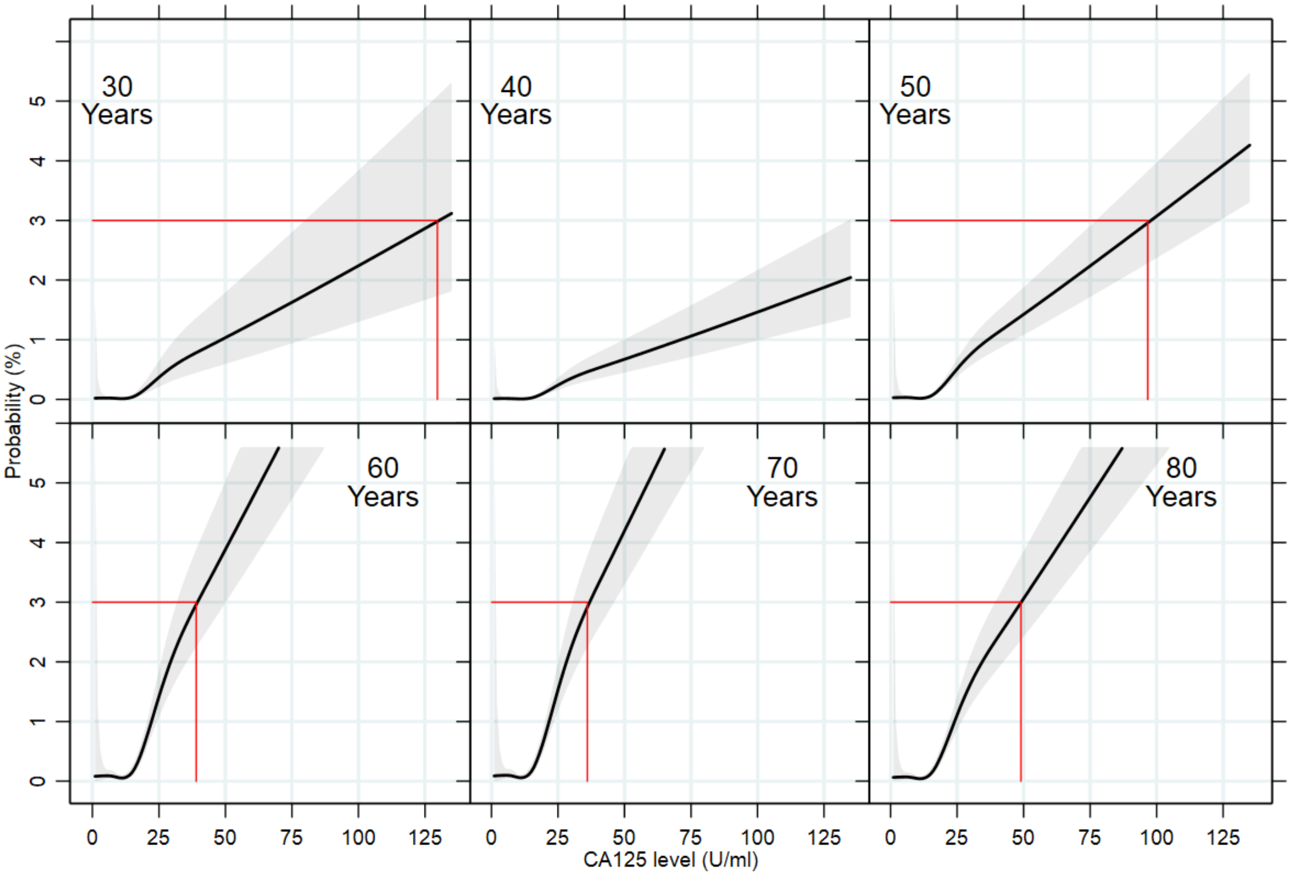

Probabilities are shown in relation to CA125 level for women of 30, 40, 50, 60, 70 and 80 years of age. CA125 levels which correspond to the closest integer probabilities of 3% are indicated in red (not displayed in 40 years of age- reached at 191 U/ml). 95% confidence intervals are displayed.
