# Supplementary material for: The diagnostic performance of CA125 for the detection of ovarian and non-ovarian cancer in primary care: A population-based cohort study
Source: PLoS Med. 2020 Oct 28;17(10):e1003295. doi: 10.1371/journal.pmed.1003295 (PMC7592785; doi:10.1371/journal.pmed.1003295)
Supplement: Attachment — Submitted filename: Editor and reviewer responses.docx [file pmed.1003295.s015.docx]

We are very grateful to the editors and reviewers for taking the time to review our manuscript. We feel that the constructive feedback we received has really helped us to improve it. Please find below a table with our response to each comment. Page and line numbers refer to locations in the ‘clean’ manuscript rather than the ‘marked-up’ manuscript.

| **Editor / reviewer** | **Editor / reviewer comment** | **Author response and revisions** |
| --- | --- | --- |
| Editors | In the abstract, we'd ask the authors to restructure this using the PLOS Medicine headings (Background, Methods and Findings, Conclusions) - "Methods and Findings" is a single subsection. | Updated (page 2) |
|  | In the last sentence of the Abstract Methods and Findings section, please describe some of the key limitation(s) of the study's methodology. | Added (page 2, line 59-62) |
|  | We ask that you include a short, non-technical Author Summary of your research to make findings accessible to a wide audience that includes both scientists and non-scientists. | Added (page 3, line 72-96 and page 4, line 97-99) |
|  | Please reformat the in-text reference callouts to use the PLOS Medicine style | Updated |
|  | Did your study have a prospective protocol or analysis plan? Please state this (either way) early in the Methods section  In either case, changes in the analysis-- including those made in response to peer review comments-- should be identified as such in the Methods section of the paper, with rationale. | The prospective protocol, submitted when obtaining regulatory approval, has been added to the supplementary material (S1 Appendix, S2 Appendix) and referenced early in methods (page 7, line 192-194).  This protocol covers several linked studies on differing aspects of ovarian cancer diagnosis. The additional linked studies covered in the protocol are currently underway and will be published in due course.  We have highlighted in the methods that the additional analysis (age as a continuous variable) was performed in response to peer review comments (page 9, line 283). All other analyses were pre-specified. |
|  | The two subsections at the very end of the paper, 'What is already known on this topic' and 'What this study adds' should be deleted | These have been removed |
|  | We'd suggest the authors include as supporting information the completed checklist for just one of these, ideally STARD as that would be the relevant guideline for the overall framework of the study | Completed STARD checklist included in supporting information (S3 Appendix). |
| Reviewer 1 | Give numbers for stage II and III | Updated to give survival for each stage (page 5, line 132-133) |
|  | Model building: The sentence starting "The number of polynomial terms ...."   Whether to use splines or polynomials is an interesting question. Using AIC to decide is one reasonable way. I tend to like splines, but polynomial terms can also work. But I would set an upper limit on the order of the polynomial (maybe cubic but no higher) because beyond that they lose the main advantage over splines - interpretability.   Please list the terms used here (and give details in the appendix). | Following the introduction of age as a continuous variable (see next point) we found that polynomials performed far less well than splines for age, whereas for CA125 level the difference was marginal. Given this, we have switched to use splines instead of polynomials and have updated the methods section (page 9, line 272-277) accordingly. We have listed the number of knots used in the text and given full model specifications in the supplementary material (S5 Appendix). |
|  | Rather than dichotomize age, leave it continuous and investigate it the same way you did CA125.  Then include the terms (spline or polynomial) in the model. This would give a probability for every combination of age and CA. And the plts could be changed to trellis plots. | We agree that treating age as a continuous variable would allow for more individualised cancer probability estimates, which is a key aim of this study. We have performed the additional analyses, in line with the reviewer’s advice, and have updated the relevant sections of the manuscript (*abstract*: page 2 line 48-49 and 58-59, *methods:* page 9, line 283 + page 10, lines 284-289, *results:* page 16, line 439-445 + Figure 3, *Discussion:* page 17 lines 463-465 + page 18, lines 492-497 + page 19, lines 523-529).  We have retained the original analysis (<50 years and ≥50 years) as we prespecified that we would look at test performance in these groups, but have relegated the figure on predicted probabilities in women <50 years and ≥50 years to the supplementary material (S10 Appendix) and restricted our description in the results to a single sentence (page 16, line 434-436). |
|  | Section on cutoff:  Maybe add a discussion of why 3% was chosen and the costs of the different errors. | We used the 3% cut-off as this is the criterion for urgent cancer referral set by NICE - the UK guideline producing body (page 6, line 164-165). There is evidence that many patients would opt for investigation at risk levels as low as 1% - we highlight that clinicians may wish to request investigations at lower cancer probabilities after discussion with their patient (page 20 line 578-580). We have added a short paragraph discussing the potential implications of using higher/lower probability cut-offs (page 21, line 587-595). |
|  | figure 2 is too small to read, at least on a printout. | This figure has been revised, with graphs for women <50 years and ≥50 years moved to the supplementary material (S10 Appendix). |
| Reviewer 2 | The only critique is that the first paragraph of Abstract is very confusing and should be revised. | We have updated the abstract slightly to improve readability (page 2). |
| Reviewer 3 | The authors note that a limitation of this study was that only contains patients being evaluated in the English system. This limitation should be expanded upon at minimum in the discussion and potentially in the results. For instance, there several confounding factors that contributes to CA125 levels including race, smoking, hysterectomy, menstrual cycle, etc. (PMID: 11352859). Changes in menstrual cycle can account for CA125 change up to 22%.  It is unclear from the manuscript whether beyond age these factors contributed or detracted from the findings. | We have expanded upon this in the discussion section, with reference to the paper highlighted by the reviewer (page 17, lines 479-485).  We did not include variables such as smoking status and hysterectomy in our analysis as our aim was to develop simple models which would allow the probability of cancer to be calculated and reported alongside the CA125 level, without the need to collect further detailed information from patients on their medical history, lifestyle etc. |
|  | Different histological subtypes for ovarian cancer should be noted, because different histotypes are not likely to have the same CA125 threshold. It is interesting that in women <50 y.o. required a CA-125 level of 179 U/mL to equate to a 3% probability of invasive ovarian cancer, which is likely due to different (other than high grade serous) and less invasive histotypes being more prevalent in pre-menopausal women. | Previous studies have found that CA125 levels do vary by ovarian tumour histology. For example, fewer patients with mucinous tumours have raised CA125 levels when compared to other epithelial ovarian tumour types. In our study, a greater proportion of cancers in <50 year old women were mucinous and non-epithelial in origin.    While, at a primary care level, the key question is whether the patient has/has not got cancer (regardless of histology), we agree that differences in histology between the age groups are worth highlighting in the paper as they aid interpretation of CA125 performance. We have included a breakdown of tumour type by age (S7 Appendix) and updated the methods (page 9, lines 256-257), results (page 11, line 330-333) and discussion (page 19, line 537-540). |
|  | Figure 2 is difficult to read, the text is too small, and the y-axis different for each graph. | This figure has been revised, with graphs for women <50 years and ≥50 years moved to the supplementary material (S10 Appendix). |
